# Altitudinal variation of microplastic abundance in lakeshore sediments from Italian lakes

**DOI:** 10.1007/s11356-024-33648-5

**Published:** 2024-05-14

**Authors:** Marco Parolini, Elena Perin, Beatrice De Felice, Stefano Gazzotti, Adriano Palazzi, Luca Conti, Eleonora Conterosito, Emanuela Rosio, Francesco Bruno, Valentina Gianotti, Roberto Cavallo

**Affiliations:** 1 Department of Environmental Science and Policy, University of Milan, Via Celoria 26, 20133 Milan, Italy; 2 Department of Sustainable Development and Ecological Transition, University of Piemonte Orientale, Via T. Michel 11, 13100 Vercelli, Italy; 3 Department of Chemistry, University of Milan, Via Golgi 19, 20133 Milan, Italy; 4 ERICA Soc. Coop, Via Santa Margherita, 26, 12051 Cuneo, Italy

**Keywords:** Microplastics, Sediments, Freshwater, Mountain

## Abstract

Microplastic (MP) contamination represents an issue of global concern for both aquatic and terrestrial ecosystems, but only in recent years, the study of MPs has been focused on freshwaters. Several monitoring surveys have detected the presence of a wide array of MPs differing in size, shape, and polymer composition in rivers and lakes worldwide. Because of their role of sink for plastic particles, the abundance of MPs was investigated in waters, and deep and shoreline sediments from diverse lakes, confirming the ubiquity of this contamination. Although diverse factors, including those concerning anthropogenic activities and physical characteristics of lakes, have been supposed to affect MP abundances, very few studies have directly addressed these links. Thus, the aim of the present study was to explore the levels of MP contamination in mountain and subalpine lakes from Northern Italy. Fourteen lakes dislocated at different altitudes and characterized by dissimilar anthropic pressures were visited. Lakeshore sediments were collected close to the drift line to assess MPs contamination. Our results showed the presence of MPs in lakeshore sediments from all the lakes, with a mean (± standard deviation) expressed as MPs/Kg dry sediment accounting to 14.42 ± 13.31 (range 1.57–61.53), while expressed as MPs/m^2^, it was 176.07 ± 172.83 (range 25.00–666.67). The MP abundance measured for Garda Lake was significantly higher compared to all the other ones (*F*_1,13_ = 7.344; *P* < 0.001). The pattern of contamination was dominated by fibers in all the lakes, but they were the main contributors in mountain lakes. These findings showed that the MP abundance varied according to the altitude of the lakes, with higher levels measured in subalpine lakes located at low altitudes and surrounded by populated areas.

## Introduction

Plastics are organic materials composed of polymers formed by the polymerization of single monomers organized into long and complex chains (Andrady 2017). Because of their peculiar physical–chemical features, including strength, flexibility, durability, and low production cost, the production and use of plastics have globally increased together with the consequent ever-expanding range of uses and applications in diverse sectors (e.g., medical, health, automotive, building, and food packaging) (Anagnosti et al. 2021). To support the increasing plastic demand, approximately 400 million tons of plastic are produced every year, with an increasing trend that is expected to double by 2050 (PlasticsEurope 2022). Despite the undeniable benefits of plastics (Andrady and Neal 2009), their improper disposal and the mismanagement of single-use or unusable plastic objects at their end-life, coupled with their persistence, resulted in their accumulation into the environment. Therefore, plastics can experience weathering processes mediated by mechanical erosion, physical abrasion, solar radiation, and biological degradation (De Sá et al. 2018), which lead to their degradation, breakage, and fragmentation in small-sized (i.e., debris) items. According to its size, plastic debris was grouped in four categories, namely nano- (1 to < 1000 nm), micro- (1 to < 1000 μm), meso- (1 to < 10 mm), and macroplastics (1 cm and larger) (Hartmann et al. 2019). In recent years, plastic contamination has raised concern because of its widespread diffusion and potential impacts on ecosystems (see Bucci et al. 2020) and human health (Wright and Kelly 2017; Alabi et al. 2019). Several studies have documented the presence of differently sized plastic debris in terrestrial and aquatic ecosystems worldwide (see Napper and Thompson 2023). Particular attention has been focused on small-sized items, mainly microplastics (MPs), which emerged as a hot-topic in environmental studies. Microplastics of diverse shape, color, size, and polymer composition have been detected in the atmosphere, as well as in diverse domains of marine ecosystems, freshwaters (i.e., rivers and lakes), soil, and biota, from both anthropic and natural ecosystems (Peng et al. 2020; Li et al. 2020; Yu et al. 2022; Yang et al. 2022; Parolini et al. 2023). Moreover, recent monitoring surveys have identified the presence of MPs also in the so-called remote and pristine ecosystems (Citterich et al. 2023; Forster et al. 2023), confirming that no physical and/or geographical barriers have limited the diffusion of this contamination. Indeed, MPs have been detected in the deep sea (Kane and Clane 2019), Arctic and Antarctica (Bergmann et al. 2022; Rota et al. 2022), waters (Free et al. 2014; Godoy et al. 2022) and sediments from high-mountain lakes (Tsering et al. 2022; Pastorino et al. 2023), snow (Parolini et al. 2020a, b; Bergmann et al. 2019), mountain soils (Padha et al. 2022), as well as glaciers (Ambrosini et al. 2019; Crosta et al. 2022).

While the study of plastic contamination in marine environments has started many years ago (Carpenter and Smith 1972; Colton et al. 1974), the focus on freshwaters is a relatively recent phenomenon, encompassing approximately the last 15 years (Talbot and Chang 2022). The investigation of plastic and MPs, contamination in freshwaters represents a crucial step in the understanding of their environmental fate. In fact, freshwaters can be the source (e.g., wastewater treatment plants), the transferring media (e.g., rivers), and the sink (e.g., lakes) of MPs (Li et al. 2018). Several monitoring studies have identified the presence of MPs in water, deep and shoreline sediments, and biota from different freshwater ecosystems worldwide (Li et al. 2018; 2020; Talbot and Chang 2022). Lakes represent crucial freshwater ecosystems for our society because of the different ecosystem services they offer, including control (i.e., flooding and drought), provision (i.e., drinking water, food production via fisheries, aquaculture and irrigation of agricultural fields, and energy production through hydropower dams), and cultural (i.e., recreation) services (Talbot and Chang 2022). Thus, the monitoring of lake contamination is pivotal to preserve water quality and related services. Concerning MP contamination, lakes act, at least temporarily, as a sink for plastic particles (Imhof et al. 2013). MPs constantly migrate and undergo several modifications in lakes, exerting impacts on ecosystems at all the levels of ecological hierarchy, including pollution diffusion and biological toxicity (Yin et al. 2023). Recent reviews has summarized the MP contamination in terms of abundances, morphologies, and polymer types in the water and sediments of lakes located in different countries and regions. The abundance and characteristics of MPs were found to largely differ across countries and regions, depending on local development level and economic structure (Yang et al. 2022). Moreover, MP abundance resulted as negatively related with the depth and the area of lakes, and the distance to populated areas, while a positive relationship with the surrounding population density was pointed out (Pan et al. 2023).

Among lakes, mountain, and specifically high-mountain, lakes are supposed to be less affected by local anthropogenic impact, such as pressures from industry, agriculture, and wastewater, which typically affect lakes at lower altitudes (Ebner et al. 2022; Pastorino et al. 2022a,b). However, diverse signs of physical–chemical, biological, and morphological alteration occur also in these ecosystems (Pastorino and Prearo 2020). High-mountain lakes are indicators of environmental issues of global concern, including deposition of acidic substances from the atmosphere, medium- and long-range transport of diverse contaminants and climate change (Moser et al. 2019; Pastorino et al. 2022b). Recent studies showed that also mountain and high-mountain lakes suffer MP contamination (Pastorino et al. 2022b and reference therein), confirming their role in determining the distribution processes and pathways, as well as the fate of MPs.

While diverse factors, including those relating to anthropogenic activities and physical characteristics of lakes, have been supposed to affect MP abundances, very few studies have directly addressed these links (Talbot and Chang 2022). Some studies have shown that the degree of MP contamination is related to the population density and urbanization; as the population density decreases with the increase of the altitude, the same is for the intensity of human activities (Lang et al. 2022). Thus, the degree of MPs contamination in high altitude areas has been demonstrated to be relatively low in rivers (Jiang et al. 2019; Wang et al. 2018), while abundances of MPs in soils have been observed being negatively correlated with the altitude (Feng et al. 2021). Despite these findings, no study has specifically focused on the role of the altitude in determining the degree of MP contamination in lakes. Thus, the aim of the present study was to explore the levels of plastic contamination, focusing on MPs, in mountain and subalpine lakes located in Northern Italy. Moreover, this study aimed at investigating the potential relationship between MP abundances and the altitude of the lakes. Indeed, although diverse studies have investigated the MP contamination in lakes located at different altitudes (see Talbot and Chang 2022, and references therein), none has explored if the altitudinal location of lakes can affect the MP abundance.

Fourteen lakes dislocated at different altitudes and characterized by dissimilar anthropic pressures were coasted during the Keep Clean and Run (KCR) event in 2023, a 350-km-long race representing the launch event of the European campaign Let’s Clean Up Europe (Cavallo 2015). Lakeshore sediments were collected at the drift line in each lake and were analyzed to assess altitudinal trends and factors determining MP contamination. Lakeshore sediments were selected as environmental matrix to assess the presence of MPs because the collection and the analysis of beach sediments from the shorelines of the sea, lakes, and streams are common practices to examine the contamination of a water body (Van Cauwenberghe et al. 2015; Ivleva et al. 2017). The advantage to analyze MPs in sediments is related to the lower particle size detection limit, which is only determined by the sample preparation and identification methods applied (Imhof et al. 2018). According to the current literature, we expected that MP abundance was positively related with the extent of anthropic activities in the surroundings of the lakes, while we had no clear a priori expectation on the relationship between MP contamination and the altitude of the lake.

## Materials and methods

### Study area and sampling design

**Fig. 1:**
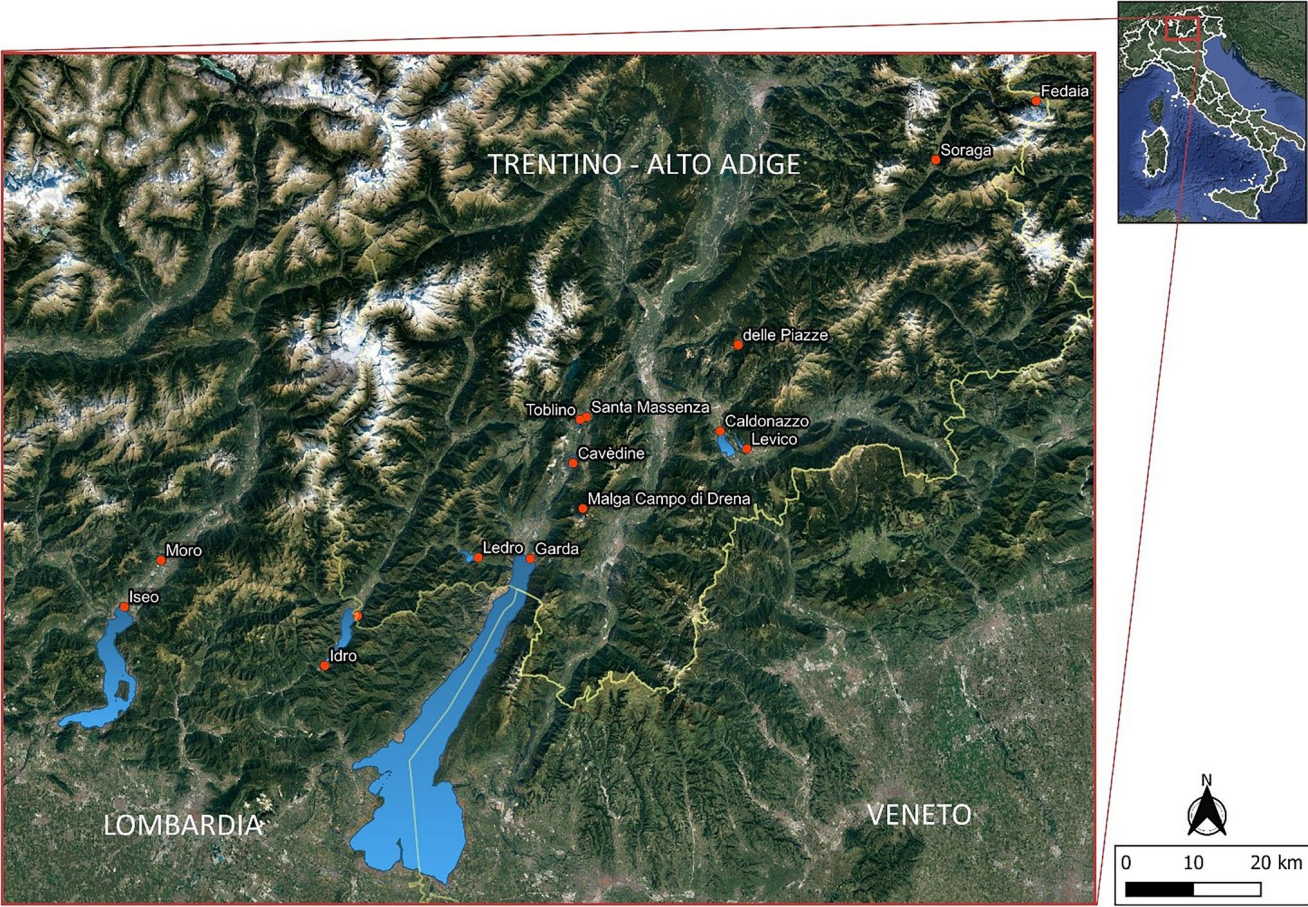
Map of sampling locations over the path travelled during the Keep Clean and Run (KCR) event in 2023

Recreational trail running and participation in organized events increased their popularity worldwide, growing at a yearly rate of 15% over the past decade (World Athletics 2022). On one hand, these events might represent a source of large-sized and small-sized plastic items into the environment because of deliberate or unintentional release of food packaging and technical equipment, as well as because the abrasion of technical clothes and running shoes during the event (Forster et al. 2023; Parolini et al. 2021). On the other hand, these sporting events represent a unique opportunity to collect samples to study plastic contamination and to raise public awareness on this environmental issue, so that real institutional agreements have even been signed to reduce the impact of such events (e.g., the International Charter of sustainable sporting events, known as the Courmayeur Charter—https://www.mase.gov.it/comunicati/il-ministero-firma-la-carta-di-courmayeur-gli-eventi-sportivi-sostenibili). The Keep Clean and Run (KCR) event is a plogging eco-marathon that since 9 years aims at cleaning up paths from abandoned waste and raising the awareness of citizens about the importance of environmental protection (https://keepcleanandrun.com/). During the 2019 edition, a companion activity focused on the investigation of MP contamination in the surface waters of the Po River was performed (Cavallo et al. 2020), confirming the role of this event in collecting samples and returning scientific data for MP monitoring. In 2023, the KCR event was run between April the 29th and May the 5th (seven stages) in Northern Italy, crossing three Italian regions (i.e., Trentino-Alto Adige, Veneto, and Lombardy). Over the KCR path, the runners coasted 14 alpine and subalpine lakes (Fig. 1), where trained operators collected lakeshore sediments to assess MP contamination. The lakes encountered over the path during the 7 days of the event were: Fedaia, Soraga, delle Piazze, Levico, Caldonazzo, Santa Massenza, Toblino, Cavèdine, Malga Campo di Drena, Garda, Ledro, Idro, Moro, and Iseo Lake. Sampling locations into each lake were selected according to the accessibility of the beaches and the presence of potential sources of contamination (e.g., inlet of rivers, roads, towns, or cities). Three sampling locations were identified in each lake (Table 1). According to the results of a previous study by Imhof and coauthors (2018), sampling was performed close to the drift line, where the higher abundance of plastic debris is expected.

**Table 1:** Geographical localization of lakes coasted during the KCR event and geolocalization of sampling sites where lakeshore samples were collected. Latitude and longitude refer to the coordinates of the sampling point of each single replicate collected from each lake

Stage (S)	Date	Municipality (province)	Lake name	Lake ID	Lake altitude (m a.s.l.)	Sample ID	Latitude	Longitude
S1	29/04/23	Canazei (TN)	Fedaia	L1	2053	R1	46.462853	11.863955
						R2	46.46284	11.864401
						R3	46.46281	11.864700
	29/04/23	Soraga di Fassa/Moena (TN)	Soraga	L2	1190	R1	46.390506	11.666178
						R2	46.389402	11.666466
						R3	46.388935	11.667271
S2	30/04/23	Bedollo (TN)	Piazze	L3	1021	R1	46.151085	11.274271
						R2	46.149776	11.27491
						R3	46.150629	11.276031
S3	01/05/23	Levico Terme (TN)	Levico	L4	440	R1	46.012684	11.286916
						R2	46.012321	11.286843
						R3	46.011707	11.286403
	01/05/23	Caldonazzo (TN)	Caldonazzo	L5	449	R1	46.036429	11.235855
						R2	46.036428	11.23578
						R3	46.036504	11.236531
S4	02/05/23	Vallelaghi (TN)	Santa Massenza	L6	250	R1	46.059532	10.977952
						R2	46.060188	10.979954
						R3	46.060037	10.981145
	02/05/23	Madruzzo (TN)	Toblino	L7	245	R1	46.056105	10.967385
						R2	46.056367	10.967740
						R3	46.056635	10.968381
	02/05/23	Cavèdine (TN)	Cavèdine	L8	241	R1	45.999920	10.953136
						R2	45.9996	10.953171
						R3	45.998834	10.953051
	02/05/23	Drena (TN)	Malga Campo di Drena	L9	1368	R1	45.938017	10.969694
						R2	45.938096	10.969678
S5	03/05/23	Nago-Torbole (TN)	Garda	L10	65	R1	45.872821	10.867412
						R2	45.872587	10.866894
						R3	45.872293	10.867058
	03/05/23	Molina di Ledro (TN)	Ledro	L11	655	R1	45.875953	10.767238
						R2	45.876189	10.767628
						R3	45.875942	10.767470
S6	04/05/23	Lemprato (BS)	Idro	L12	368	R1	45.735810	10.468725
						R2	45.736277	10.469610
						R3	45.735944	10.470306
	04/05/23	Baitoni (TN)	Idro	L12	368	R1	45.801977	10.534700
						R2	45.802222	10.534519
						R3	45.801448	10.534722
S7	05/05/23	Darfo Boario Terme (BS)	Moro	L13	380	R1	45.880357	10.162482
						R2	45.880157	10.162105
						R3	45.879970	10.160903
	05/05/23	Costa Volpino (BG)	Iseo	L14	185	R1	45.819022	10.090035
						R2	45.818766	10.089744
						R3	45.818657	10.089435

Lakeshore sediments were collected with a stainless steel shovel preliminarily washed with acetone. The shovel was washed between the sampling of each single sample. After the identification of the sampling location, the specific sampling point was randomly selected through a quadrat sampling approach. A 20 × 20 cm plastic square (0.04 m^2^), wrapped with tinfoil to avoid the potential release of MPs, was blindly thrown on the beach. Then, the lakeshore sediments (the first 2–3 cm in depth of the surface sediments of the beach) were collected with a stainless steel shovel and transferred to 500-mL glassware jars. Jars were previously washed with acetone, and their opening was sealed with a tinfoil to avoid external contamination. All samples were named reporting on the label the number of the KCR stage (S 1–7), the progressive identification of the lake (L 1–14), and the number of the sample (i.e., replicate) collected in each basin (R 1–3), as well as the sampling date. Sediments were maintained at 4 °C until the transfer in the lab, where they were dried into an oven at 60 °C for 48 h. After desiccation, sediments were processed to isolate MPs.

### Isolation and characterization of MPs

All the glassware, stainless forceps, and pins used during the laboratory procedure to isolate MPs were preliminarily washed with acetone and ultrapure water filtered on cellulose filters (StonyLab, pore size 1 m; *Ø* = 46 mm). Then, they were wrapped in a tinfoil to avoid external contamination until processing the sediments. A total of 44 lakeshore sediment samples were collected, corresponding to three independent samples (with the exception of Malga Campo di Drena Lake where only two samples were collected because of the very small lake size) from the 14 alpine and subalpine lakes coasted during the KCR event. Moreover, in the Idro Lake lakeshore, sediment samples were collected in two different places of the lake (one in the territory of Trentino-Alto Adige and one of the Lombardy regions), for a total of six samples.

MPs were isolated from sediments through a previously validated method (see Ambrosini et al. 2019; Crosta et al. 2022), with slight modifications. Sediments from each lake were weighted and 265 ± 126 g (mean ± standard deviation; range 17–501 g, depending on the amount of the sample) were included in beaker added with 300 mL of saturated NaCl solution (density = 1.2 g/cm^3^). The solution was first mixed using a metal spoon and then stirred for 15 min. Then, it was covered with tinfoil and left decanting at room temperature overnight. A batch of six samples was processed at the same time, and it was flanked by a control sample made of 300 mL NaCl solution only that was processed as the environmental samples. The day next, digestion of organic matter was performed using the Fenton’s reagent (see Prata et al. 2019). The upper phase of the solution (ca. 200 mL) was transferred to another beaker, which was added with 20 mL of the Fenton’s reagent (FeSO_4_) and 20 mL of hydrogen peroxide (30% v/v, previously filtered on cellulose filters). After 5 min, an additional volume of 20 mL of H_2_O_2_ was added. The solution was covered with a tinfoil, heated in the oven for 1 h at 50 °C, and then left at room temperature overnight. The control sample underwent the same procedure. Each sample was filtered through a lab vacuum filtration apparatus on cellulose filter (StonyLab, pore size 1 µm; *Ø* = 46 mm). Each filter was then kept in a glass petri dish (*Ø* = 50 mm). The beakers were rinsed twice with 20 mL of filtered ultrapure H_2_O to collect any eventual MPs adsorbed on the glassware. Whenever large-sized plastic items (larger than 1 mm) were detected, they were transferred to another petri dish with forceps and categorized as mesoplastics.

According to the procedure described by Nava et al. (2023), a preliminary visual inspection (according to shape and color of items) of each filter was performed under a stereomicroscope (Leica EZ4W). Every item that was putatively identified as a MP was picked up with a stainless pin and transferred to a silver metal membrane filter (Sterlitech, pore size 0.8 µm; *Ø* = 13 mm) until polymer characterization. A picture of each filter was captured to allow the measurement of size (expressed as the maximum length of item) of each item with the image processing package Fiji freeware software (Schindelin et al. 2012).

### *Quality control and assurance*

In order to check for potential contamination of the laboratory, a cellulose filter was place on a tinfoil close to the equipment used during the analytical procedure (Parolini et al. 2021). Moreover, in order to assess the efficiency of the extraction and purification methodology used in the present study, a mass recovery test was performed according to the procedure described in detail by Winkler et al. (2022). As the most of polymers found in freshwater environment are made of polyethylene, polypropylene, and polyester (mainly PET), we decided to focus our recovery tests on microplastics made of polyethylene (PE), the polypropylene (PP), and the polyethylene terephthalate (PET). Moreover, PE and PP can be considered as low-density polymers (range of density 0.88–0.96 g/cm^3^ and 0.895 and 0.92 g/cm^3^, respectively), while PET can be considered a high-density polymer (1.38 g/cm^3^). Twenty (20) mg of MPs made of PE, PP, or PET were spiked into 30 g of aquarium sand. Microplastics were obtained from a series of freezing and grinding cycles of objects made of PE, PP, and PET according to the procedure described elsewhere (Parolini et al. 2020a,b). Microplastics were then sieved to < 1000 μm. Spiked sand samples were processed according to the same procedure described above. Three replicates per each polymer were performed. The mean (± SD) recovery rate for PE-, PP-, and PET-MPs was 96.4 ± 2.6%, 89.2 ± 11.2%, and 82.2 ± 6.9%, respectively.

### Polymer characterization

The polymer characterization of putative MPs was performed through Fourier transformed infrared (FTIR) microscopy (µ-FTIR), using a Nicolet iN10 MX Infrared Imaging Microscope (Thermo Scientific, Waltham, MA, USA). The characterization was performed in reflection mode in a wavenumber range of 4000–650 cm^−1^. OMNIC™ Picta software (Thermo Scientific, Waltham, MA, USA) controlled the instrument. A total of 256 scans were acquired for each spectrum, with a spectral resolution of 4 cm^−1^. Different libraries were used for polymer identification, including HR Aldrich Polymers, HR Coatings Technology, HR Hummel Polymer and Additives, HR Industrial Coatings, HR Polymer Additives and Plasticizers, HR Rubber Compounding Materials, HR Spectra Polymers and Plasticizers, Hummel Polymer sample Library, and Polymer Laminate Films. While all the fragments were processed under µ-FTIR, only the 25% of fibers were randomly collected from the filters and characterized because of the high number we isolated from sediment samples. The polymer composition of the other fibers was assessed through the application of the fluorophore, the 1-pyrenebutyric acid N-hydroxysuccinimidyl ester (PBN). Indeed, a recent study confirmed the use of PBN as a rapid, simple, cost-effective, and highly efficient detection method for the identification of different plastic polymers (Lee and Chae 2021).

### Statistical analysis

Differences in MP contamination of lakeshore sediments were assessed through the application of a one-way analysis of variance (ANOVA), after checking for normality and homoscedasticity of data by means of Shapiro–Wilk and Levene’s tests, respectively. Pairwise differences of MP contamination between lakes were checked through the Tukey’s post-hoc test. Significance was set at *P* < 0.05 (*) and *P* < 0.01 (**). A linear regression analysis was performed to assess the relationship between the altitude of the lakes and the MP contamination. All the statistical analyses were run in R 4.03 using the *lmer4* package (R Core Team 2020).

## Results

**Fig. 2:**
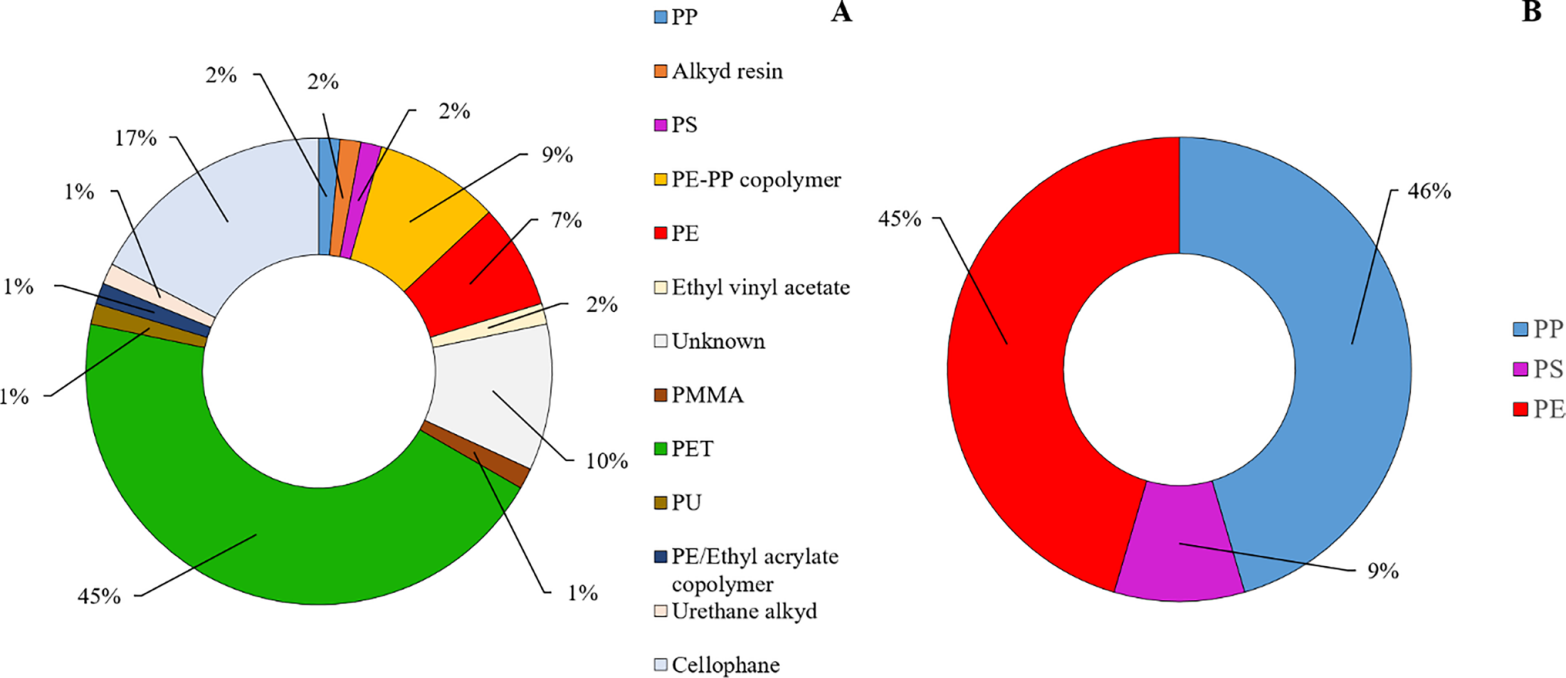
Polymer composition of microplastics (**A**) and mesoplastics (**B**) isolated from lakeshore sediments from the 14 lakes coasted during the KCR event. PP, polypropylene, PS, polystyrene, PE, polyethylene, PMMA, polymethyl methacrylate, PET, polyethylene terephthalate, PU, polyurethane

No item was found neither in procedural blanks nor on filters used to check for aerial contamination of the laboratory. Following preliminary visual inspection, putative MPs were isolated from all the analyzed samples. Overall, independently of the lake, 135 items putatively identifiable as MPs were isolated from lakeshore sediments, 95 of them (70%) were fibers, while the remaining part were fragments. The amount (mean ± standard error) of fibers (0.78 ± 0.25) was significantly higher compared to fragments (2.42 ± 0.31) (paired Student’s *t* test: *t* =  − 3.845; *P* < 0.001). All the isolated fragments and the 60% of fibers were identified through µ-FTIR analysis as MPs, while only the 46% of the fibers analyzed with the PBN. Overall, coupling both the characterization methods, the 53% of the fibers isolated from our samples were made of plastic polymers. The size of MPs, in terms of the maximum length of each single item (including both fibers and fragments), ranged between 74 and 980 μm, with a mean (± standard deviation) size of 543 ± 174 µm. Focusing on fragments only, the mean size was 180 ± 76 µm (range 74–339 µm), while for fibers, 907 ± 221 µm (range 297–980 µm). The main color of MPs was black (35%), followed by blue (22%), white/transparent (17%), and red (15%). Polyethylene terephthalate (45%) was the most abundant polymer, followed by cellophane (17%), polyethylene-polypropylene copolymer (9%), and polyethylene (7%). The 10% of isolated items were not referred to a specific plastic polymer, so they were included in a category named “Unknown” (Fig. 2A). We also isolated 12 fragments and two fibers attributable to mesoplastics from lakeshore sediments collected in five lakes only (i.e., Fedaia, Soraga, Caldonazzo, Garda, and Iseo Lakes; 1 to 3 items per lake), whose mean (± standard deviation) size was 78 ± 13 mm. The main colors were transparent (26%), red (13%), and green (9%), and they were mainly made by polypropylene (46%) and polyethylene (45%) (Fig. 2B).

**Fig. 3:**
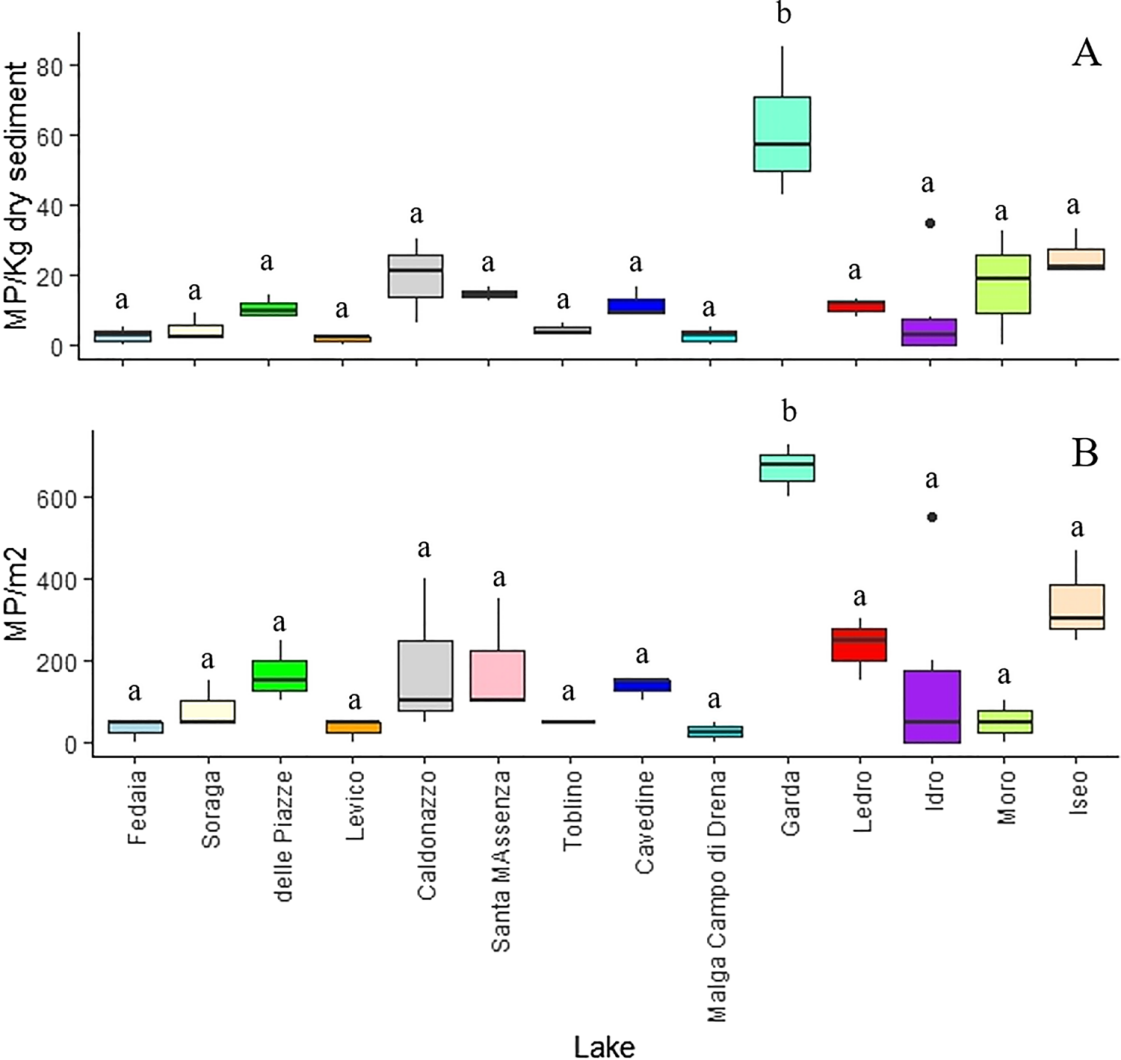
Box plots report the mean (± standard deviation) of MP contamination (expressed as MPs/Kg dry sediment (**A)** and MPs/m.^2^ (**B**)) in lakeshore sediments from the 14 lakes coasted during the KCR event. Black dots represent MP levels measured in each single replicate from all the lakes. Letters above the box plots indicate statistical differences among MP contamination in lakes. Different letters indicate significant differences (*P* < 0.05)

The mean (± standard deviation) MP contamination level expressed as MPs/Kg dry sediment was 14.42 ± 13.31 (range 1.57–61.53), while expressed as MPs/m^2^, it was 176.07 ± 172.83 (range 25.00–666.67). The highest abundance of MPs expressed as MPs/Kg dry sediment (Fig. 2) was found in lakeshore sediments from the Garda Lake (mean ± standard error: 61.53 ± 12.38 MPs/Kg of dry sediment), followed by Iseo (25.40 ± 3.79 MPs/Kg of sediment), Caldonazzo (19.01 ± 7.03 MPs/Kg of dry sediment), Moro (16.92 ± 9.34 MPs/Kg of sediment), and Idro (15.96 ± 9.51 MPs/Kg of dry sediment) lakes. In contrast, very low levels of MPs were identified in lakes located at higher altitudes and characterized by a lower anthropic attendance, such as Fedaia (2.54 ± 1.43 MPs/Kg of dry sediment) and Malga Campo di Drena (2.52 ± 2.06 MPs/Kg of dry sediment) lakes (Fig. 3).

The statistical analysis showed that the MP contamination significantly differed among lakes (*F*_1,13_ = 7.344; *P* < 0.001). Specifically, the MP lakeshore contamination observed in the Garda Lake was significantly higher compared to that of all the other ones (*P* < 0.005 for all the pairwise comparisons). In contrast, no significant pairwise differences were noted on the MP contamination in the other lakes. Qualitatively and quantitatively similar results were obtained considering the MP contamination expressed as MPs/m^2^ (*F*_1,13_ = 4.748; *P* < 0.001; Fig. 3B). As for the contamination expressed as MPs/Kg dry sediment, the highest levels were found in lakeshore sediments from the Garda (mean ± standard error: 666.66 ± 36.32 MPs/m^2^), followed by Iseo (340.00 ± 66.58 MPs/m^2^) lake.

**Fig. 4:**
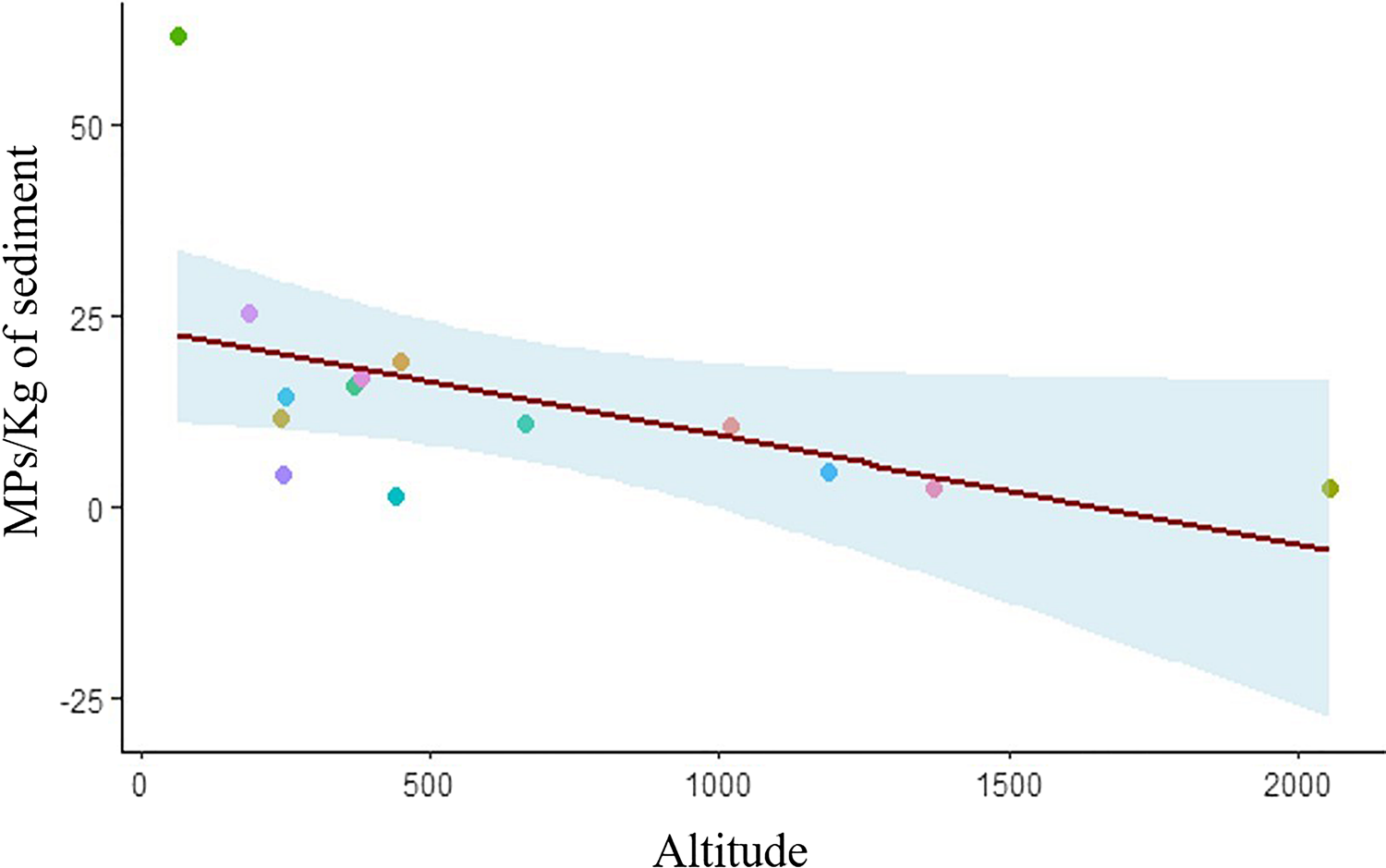
Relationship between the MP lakeshore sediment contamination (expressed in MPs/Kg of dry sediment) and the altitude of the lake (meters above sea levels). Each dot represents the mean MP contamination measured for each of the 14 lakes coasted during the KCR event

Interestingly, a significant relationship between the amount of MP in lakeshore sediments, expressed as MPs/Kg dry sediment, and the altitude of the lake was found (estimate: − 0.012 ± 0.004; *t* value =  − 2.894; *P* = 0.006; *R*^2^ = 0.269), whereby the MP contamination decreases at increasing the altitude of the lake (Fig. 4). A similar, but marginally non-significant (estimate: − 0.149 ± 0.076; *t* value =  − 1.952; *P* = 0.074; *R*^2^ = 0.241) relationship was found considering the MP contamination expressed as MPs/m^2^ (data not shown).

**Fig. 5:**
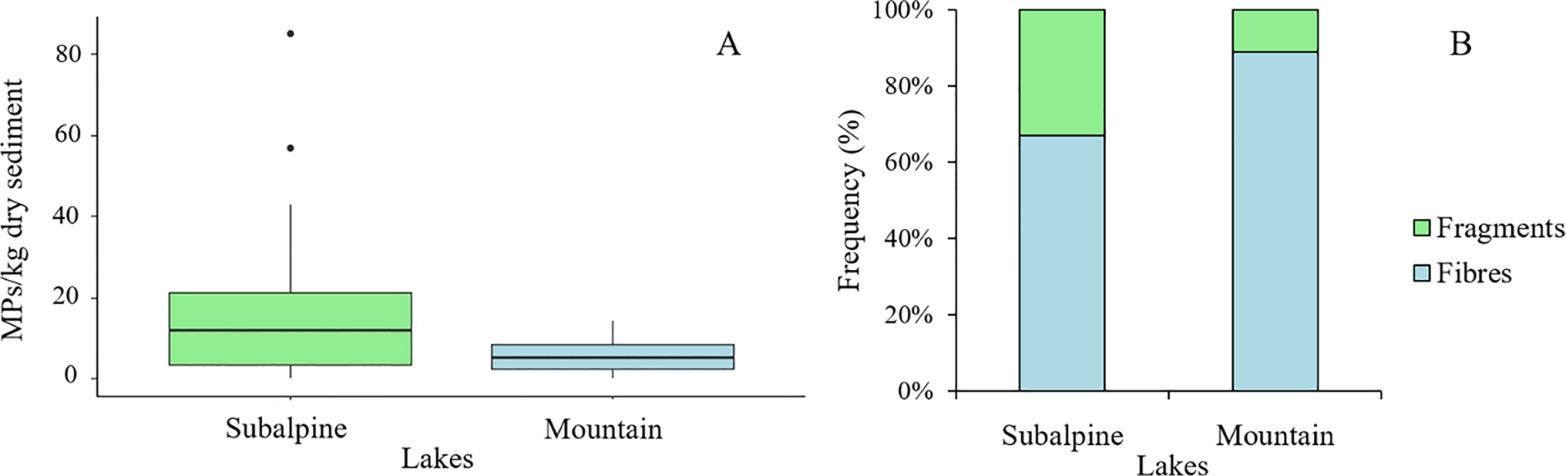
MP abundance (expresses as MPs/Kg dry sediment) (**A**) and pattern of contamination (**B**) in subalpine and mountain lakes coasted during the KCR event

Grouping lakes according to their altitude in mountain (> 1000 m a.s.l.) and subalpine (< 1000 m a.s.l.), a marginally non-significant difference was noted (*F* = 3.940; *P* = 0.053), with mountain lakes showing lower MP abundance than subalpine ones (Fig. 5A). A slight difference in MP contamination pattern (Fig. 5B) appeared; while fibers accounted for the 89% of total MPs in mountain lakes, their contribution of contamination of subalpine lakes accounted for only the 67%. However, statistical analysis did not show any significant difference in the number (*F* = 1.327; *P* = 0.256) and in the abundance (expressed as fibers/Kg dry sediment – *F* = 1.753; *P* = 0.192) of fibers between subalpine and mountain lakes occurred.

## Discussion

The present study confirmed the presence of MPs in lakeshore sediments from both mountain and subalpine lakes, showing differences in the levels and the pattern of contamination that varied according to the altitude where the lake is located.

The MP abundances measured in lakeshore sediments collected in mountain and subalpine lakes from Northern Italy were compared to those obtained in the same matrix from other lakes worldwide. The comparison was performed using MP levels expressed as MPs/m^2^ or MPs/Kg dry sediment to maximize the number of comparisons. In fact, no standardization and homogenization of the units of MP contamination are currently available. The MP contamination we observed in lakeshore sediments was in a similar range as found in other studies, although notable methodological differences occurred. For instance, MP levels observed in some Switzerland lakes, namely Constance, Geneve, Neuchatel, Brienz, and Zurich lakes, ranged between 320 ± 220 and 2500 ± 300 MPs/m^2^ (Faure et al. 2015). A similar MP abundance was detected in the beach sediments of four lakes with different distances to urban areas from the Tibet plateau (range 4–563 MP/m^2^; Zhang et al. 2016), as well as in lakeshore sediments from the China’s largest inland lake, i.e., Qinghai Lake (range 50 to 1292 MPs/m^2^; Xiong et al. 2018). Mean levels of MPs measured in two lakes from Central Italy, namely Chiusi (2117 ± 695 MPs/m^2^) and Bolsena (1922 ± 662 MPs/m^2^) lakes, were higher compared to those observed in the present study. However, considering MP contamination expressed as MPs/Kg dry sediments, the contamination resulted as similar to those observed in lakes from Northern Italy (Chiusi: 234 ± 85 MPs/Kg dry sediment and Bolsena: 112 ± 32 MPs/Kg dry sediments; Fisher et al. 2016). Moreover, the MP abundance was in the same range observed in Lake Superior (USA—0 to 55 MPs/Kg dry sediment; Minor et al. 2020), but it was lower compared to 12 lakes of Tibet (range 17–2643 MPs/Kg dry sediment; Liang et al. 2022), Lake Victoria (Uganda; range 0–1102 MPs/Kg dry sediment; Egessa et al. 2020), Anchar lakes (Northwest Himalaya; range 233–1533 MPs/Kg dry sediment; Neelavannan et al. 2022), and Tollense Lake (Northeastern Germany; 1410 ± 822 MPs/Kg dry; Henstmann et al. 2021). Focusing on the Garda Lake only, our results showed that MP abundance was lower compared to monitoring surveys conducted in 2013 (1108 ± 983 MPs/m^2^; Imhof et al. 2013) and in 2018 (3508 ± 8855 MPs/m^2^; Imhof et al. 2018). However, the comparison of the abundance of MPs on Lake Garda lakeshore sediments has to be performed with caution because of the different methodologies used for sampling, sample preparation, and particle identification (Imhof et al. 2018). Moreover, although our sampling was performed in the northern part of the Garda Lake as the previous studies, the lower MP abundance we observed could be referred to diverse physical factors considered as drivers of variation in MP abundance at beaches, including waves, water currents, wind speed, and direction (Browne et al. 2015), as well as type of sediment sampled (Hengstmann et al. 2021). All these factors are characterized by both seasonal and annual variability and, consequently, they can affect the transport, the deposition, and the isolation of MPs in lakeshore sediments.

The studies mentioned above suggested that diverse factors, including those relating to anthropogenic activities and physical features of the watershed, can affect the spatial distribution of MPs (Talbot and Chang 2022). Land cover, the presence of wastewater treatment plants, and population density were identified as the main contributors to the MP abundance in rivers and lakes (Talbot and Chang 2022). For instance, a strict relationship between MP abundance in freshwaters and urban land cover (Feng et al. 2020; Su et al. 2020; de Carvalho et al. 2021; Sang et al. 2021) was observed, with high levels of MPs identified close to urban or industrial centers (Ding et al. 2019; Luo et al. 2019; Huang et al. 2021) and in areas characterized by high population densities (see Talbot and Chang 2022 and references therein). MPs in freshwaters can originate from a broad range of terrestrial sources, including the improper and/or inefficient waste management strategies and littering (Battulga et al. 2019; Mani and Burkhardt-Holm 2020). Moreover, fibers from the laundering of synthetic materials (Peller et al. 2019) and pellets added to personal care products (McCormick et al. 2016) enter the sewage and contribute to freshwater contamination in urban areas. Our results should suggest that the higher abundance of MPs can be found in lakes close to urban and/or densely populated areas. Indeed, the highest levels of MPs were isolated from sediments collected on the shoreline of the Garda Lake, the largest subalpine Italian lake, which is characterized by larger watershed surface, population density, and touristic and anthropic activities compared to the other lakes investigated in this study.

In addition to anthropic factors, also physical watershed characteristics (e.g., elevation, slope) might affect MP abundances, although a limited number of studies addressed these relationships (Talbot and Chang 2022). For instance, high levels of MPs were found in Australian water bodies located at lower elevations compared to higher ones (Su et al. 2020). Our results confirmed that the highest MP abundances characterized lakes located at low elevations. Similar results were obtained by a previous study performed on Tibetan Plateau, where the abundance of MPs in soil resulted as negatively correlated with the altitude (Lang et al. 2022). This relationship can be related to the decrease in the intensity of human activities at different altitudes (Feng et al. 2021). Although MPs were detected also in mountain (and high-mountain lakes), the abundances measured in their sediments were lower compared to subalpine ones. The presence of MPs in mountain lakes was expected and confirmed the results of previous surveys performed in lakes from diverse geographical areas worldwide (Talbot and Chang 2022; Pastorino et al. 2023). MPs can reach mountain lakes and ecosystems through different pathways. First, MPs can enter mountain lakes as consequence of degradation and/or fragmentation of large-sized plastic items abandoned involuntarily or deliberately on shorelines mediated by physical–chemical or biological processes (Pickett 2018), as well as by the wear of clothes and mountain equipment. The intense tourist activities characterizing some mountain ecosystems and lakes can result in increased littering and, consequently, in the transfer of plastic wastes to downstream locations and/or their weathering originating MPs (Feng et al. 2020). Moreover, recreational activities, such as fishing and aquatic sports, can cause the release of differently sized plastic items, which can degrade and remain in freshwater ecosystems for long time (Alfonso et al. 2020; Xia et al. 2020). In addition to local, direct MP sources, wind and dry or wet deposition processes can serve as mid- or large-scale transport mechanism of MPs from developed regions to remote ones (Jiang et al. 2019). Recent studies demonstrated that atmospheric transport represents the main pathway of MPs to remote areas, including mountain ones (Allen et al. 2019; Wang et al. 2022). Thus, MPs transported by natural and artificial vectors contaminate the atmosphere far from areas of industrial production (Petersen and Hubbart 2021; Abbasi et al. 2023) and enter mountain soil and freshwaters through wet (for larger MPs) and dry (for small MPs and nanoparticles) deposition (Allen et al. 2019).

In addition, also the pattern of contamination slightly differed between mountain and subalpine lakes. Fibers were the predominant shape of MPs in all the lakes, independently of the altitude, as previously observed also in lakeshore sediments from other lakes (e.g., Fisher et al. 2016). In particular, higher prevalence of fibers compared to fragments was observed in mountain lakes compared to subalpine ones, although only marginally non-significant differences in the number and abundance of fibers occurred. The size distribution of MPs suggests that while the majority of fibers exhibit a small range of sizes, there are also instances of larger fibers being detected. The higher prevalence of fibers in sediments from mountain lakes can be mainly related to mid- to long-range atmospheric transport and subsequent dry and wet deposition (Allen et al. 2019; Zhang et al. 2021). Fibers are the dominant shape of MPs in the atmosphere (Chen et al. 2020), so they could reach lake sediments through deposition processes. A recent modelling study showed that microplastic fibers of about 1-mm length emitted in populated areas are more likely to reach extremely remote regions of the globe, including the high Arctic (Tatsii et al. 2023). For instance, a study by Gonzáles-Pleiter et al. (2020) showed the prevalence of fibers in a remote polar freshwater lake, suspecting that atmospheric deposition from local and afar sources was the origin of the microfibers. Other studies have proven or modelled the transport of MPs by winds, and they have indicated that the amount of MPs could vary seasonally, depending on the regional human activity and the atmospheric conditions of the region (Iwasaki et al. 2017; Allen et al. 2019). The hypothesis related to the atmospheric transport and the contribution of local human activities was also suggested by a study performed in mountain and high-mountain ecosystems. For instance, fibers prevailed in snow samples from remote areas with a very low anthropic impact, such as the Alps (Parolini et al. 2021) and the Tibetan Plateau (Zhang et al. 2021). Similarly, a large prevalence of fibers was observed in samples of supraglacial debris from two Italian glaciers characterized by a low anthropic pressure, namely the Forni and the Cedec glaciers, while fragments were the dominant shape of MPs in the Ebenferner-Vedretta Piana glacier, which suffer a higher pressure due to the presence of ski slopes (Crosta et al. 2022). The polymer composition of MPs was dominated by PET, which together with cellophane, represents the polymer composing fibers. In contrast, PE and PP were the main polymers composing MP fragments and mesoplastics found in lakeshore sediments. These results agreed previous findings showing that PP, PE, PS, and PET were the dominant polymers of MPs found in atmospheric fallout and lake sediments (Chen et al. 2020; Pastorino et al. 2023). PP and PE reflect the global demand of plastic polymers (PlasticsEurope 2022) and their extensive use in different objects of daily use, while PP and PET are commonly used to produce polyester fiber, fabric, textiles, packaging material, and reusable products. (Allen et al. 2019). In addition, the prevalence of MP fragments made of PE and PP could be due to their density lower than the water, favoring their transport by currents and deposition on sediments.

## Conclusion

Our study confirmed the widespread diffusion of MP contamination in lakeshore sediments collected in 14 mountain and subalpine lakes from Northern Italy. MP abundance varied according to the altitude of the lake, with higher levels observed in lakes at low altitudes. The levels of contamination in low altitude, subalpine lakes could be due to the closeness to urban and densely populated areas. Fibers were the main shape of MPs isolated from all the lakeshore sediments, but the higher prevalence was observed in high mountain lakes, probably due to the atmospheric transport. PET and polyolefin (PP and PE) were the main polymers composing the isolated MPs (and mesoplastics as well). Lastly, this study confirmed that sporting events are suitable opportunities to collect environmental samples for studying plastic contamination. The interaction between such activities and scientific research can contribute to raise public awareness and to develop strategies to prevent the release and accumulation of plastics in the environment. Considering our findings, further studies should be addressed to confirm the role of atmospheric transport in determining the differences in the pattern of contamination by MPs observed in high mountain lakes compared to low altitude ones. Moreover, it should be interesting to explore and confirm the relationships between MP contamination in lake and urbanization, land use, and touristic activities.

## Data Availability

Data will be made available on request.
